# Objective Image-Based Assessment of Tooth Translucency Changes Following Different Bleaching Protocols: A Retrospective Cohort Study

**DOI:** 10.3390/biomimetics11070454

**Published:** 2026-07-01

**Authors:** Ruben Pereira, João Silveira, Susana Dias, Sofia Monteiro, António Mata, Duarte Marques

**Affiliations:** 1 Oral Biology and Biochemistry Research Group, Faculty of Dental Medicine, Universidade de Lisboa, 1600-277 Lisboa, Portugal; dias.susana@edu.ulisboa.pt (S.D.); sbmonteiro@edu.ulisboa.pt (S.M.); 2 Oral Biology and Biochemistry Research Group—LIBPhys-FCT UIDB/04559/2020, Faculty of Dental Medicine, Universidade de Lisboa, 1600-277 Lisboa, Portugal; silveira@edu.ulisboa.pt (J.S.); admata2@edu.ulisboa.pt (A.M.); duarte.marques@edu.ulisboa.pt (D.M.)

**Keywords:** tooth bleaching, colour, colorimetry, aesthetics, spectrophotometry, biomimetics

## Abstract

**Background**: Tooth bleaching is a conservative aesthetic treatment that may influence tooth translucency, an optical property relevant to biomimetic dentistry. The purpose of this study is to evaluate changes in tooth translucency after three bleaching protocols, while preliminarily testing an objective, image-based, and spatially resolved method. **Materials and Methods**: A retrospective analysis used data from a previously published randomised clinical trial comparing three bleaching systems: in-office 6% hydrogen peroxide paint-on varnish; at-home 6% hydrogen peroxide prefilled tray; and at-home 16% carbamide peroxide custom tray. Spectrophotometric images of maxillary central incisors and canines were retrieved at different stages, and their translucency maps were processed with ImageJ to quantify the percentage of translucent area, histogram-derived grey intensity and RGB-blue channel metrics. Statistical tests were performed appropriately (α = 0.05). **Results**: All bleaching protocols produced significant post-treatment increases in translucency-related parameters (*p* < 0.05), although the in-office protocol showed smaller changes than the at-home techniques (*p* < 0.05). At six months, the translucent area remained significantly higher in most conditions, whereas histogram-derived metrics showed no significant changes. Correlations between translucency-related parameters and colour/whiteness differences were mostly negligible to weak. **Conclusions**: The preliminary image-based assessment detected significant and technique-dependent changes in translucency-related parameters following bleaching, with a weak linear association between these changes and colour outcomes.

## 1. Introduction

Tooth colour is a relevant topic for oral health professionals, patients, and the dental industry because it is a key determinant of aesthetic treatment outcomes [1]. The perceived colour of a tooth results from the complex interaction between the intrinsic optical properties of enamel and dentin (in combination with volume distribution) and the way light is reflected, absorbed, transmitted, and scattered within these tissues [2,3]. Therefore, from a biomimetic perspective, reproducing the natural appearance of teeth requires more than shade matching alone [4]. Because dentin is more chromatic, it primarily determines tooth hue and chroma, whereas enamel thickness, surface characteristics, and microstructure modulate light transmission and strongly influence translucency and value [5,6]. Consequently, reductions in enamel thickness increase the visibility of the underlying dentin, making its colour more perceptible as translucency and value change [7,8]. These dimensions were initially systematized in the Munsell colour system (nowadays replaced by the CIELAB system due to the lack of colour difference formulas), which standardized colour description and communication, although translucency was not originally included as a formal colour dimension [9]. Nevertheless, translucency is now regarded as a critical optical property in aesthetic dentistry and a biomimetic target, because reproducing the natural appearance of teeth requires restorative materials and clinical procedures that emulate the optical behaviour of enamel and dentin as closely as possible [7,8,10,11,12].

Tooth translucency is an optical property related to the degree of light transmission, ranging from transparency (all light transmitted) to opacity (all light absorbed or reflected) [7,10,12]. The quantification of tooth translucency is usually determined by either the translucency parameter or contrast ratio [8,13,14,15]. The contrast ratio is the ratio of the reflectance of a specimen measured over a black backing to that measured over a white backing, whereas the translucency parameter corresponds to the colour difference, of the same specimen (with uniform thickness), used to compute the difference between both conditions [8,13,14]. Recent advances in non-contact spectrophotometry allow assessment of light intensity variation across the tooth surface, thereby generating translucency maps by analysing light reflectance [16,17,18]. However, the output is typically presented on a qualitative visual scale, which limits objectivity and quantitative interpretation. Therefore, despite these technological advances, translucency parameter-based methods remain the most commonly adopted approach, since they provide a simple and convenient numerical quantification which might correspond directly to common visual assessment [8,14,19,20]. Nevertheless, these methods also have limitations due to the absence of area analysis and being based on CIE colorimetry, implying that a conditioning factor for tooth colour determination could influence translucency measurement [14].

Tooth bleaching is widely regarded as a conservative and effective aesthetic treatment option for attaining a patient’s desired smile when tooth integrity is preserved [21,22,23]. From a biomimetic perspective, however, aesthetic success depends not only on increasing whiteness, but also on maintaining the natural optical characteristics of dental tissues. Therefore, while patient demand is generally directed toward whiter teeth, a completely opaque white appearance is aesthetically undesirable, as natural teeth combine lightness with a degree of translucency [10]. However, most studies report minimal to no significant changes in translucency after tooth bleaching, which may be due to either bleaching not affecting the scattering coefficient or limitations of the current in vivo methods for translucency assessment [24,25,26,27,28]. In the study by Todorov et al. [18], a novel translucency measuring method was tested using spectrophotometric translucency maps and ImageJ 1.53 software to calculate the percentage of the vestibular surface classified as translucent. Nevertheless, no studies to date have assessed intensity-based or colour-channel histogram metrics derived from spectrophotometric translucency maps, nor have they investigated longitudinal changes after tooth bleaching.

Considering that most tooth bleaching studies using traditional contrast ratio or translucency parameter methods report inconsistent changes in tooth translucency, the availability of a reproducible, image-based, and spatially resolved approach could provide new insights into the optical effects of bleaching procedures and their biomimetic implications. Therefore, the primary aim of this study was to evaluate changes in tooth translucency following three different bleaching protocols while preliminarily testing the applicability of an objective, image-based, and spatially resolved methodology based on spectrophotometric translucency maps. The primary null hypotheses were that bleaching would not produce differences in translucency-related parameters (measured by a new pilot method) immediately after treatment or at six-month follow-up, nor between different bleaching techniques. As a secondary objective, the study aimed to investigate whether changes in tooth translucency were associated with colour and whiteness differences resulting from the bleaching procedures. The corresponding secondary null hypotheses were that no association exists between translucency changes and colour (ΔE_00_) or whiteness (ΔWI_D_) differences, either immediately after bleaching or at six-month follow-up.

## 2. Materials and Methods

This study consisted of a retrospective cohort which analysed clinical data and images derived from a previously published randomized clinical trial conducted by Pereira et al. [29]. The original trial was registered at ClinicalTrials.gov (NCT03588871) and received approval from the local Ethics Committee of the Faculty of Dental Medicine of the University of Lisbon. All procedures were performed in accordance with the Declaration of Helsinki, and written informed consent was obtained from all participants prior to enrolment. The original trial was designed to compare the bleaching efficacy and oral health-related quality of life associated with three different bleaching systems with comparable hydrogen peroxide concentrations or carbamide peroxide equivalents. Sample size calculation, randomisation procedures, and ethical approvals were fully reported in the original publication [29]. For the present study, the required data and images were retrieved to develop a pilot approach for measuring tooth translucency and to preliminarily test its applicability.

### 2.1. Study Design and Participants

The original study was designed as a three parallel groups clinical trial corresponding to different products and techniques: group A, in-office paint-on varnish 6% HP (VivaStyle Paint On Plus, Ivoclar Vivadent, Schaan, Liechtenstein); group B, at-home 6% HP with a prefilled disposable tray (Opalescence GO, Ultradent, South Jordan, UT, USA); and group C, at-home 16% CP with a customized tray (Opalescence PF 16% CP, Ultradent, South Jordan, UT, USA).

Participants who attended the dental faculty clinic were considered for eligibility and consecutively enrolled if they met the following inclusion criteria: minimum age of 18 years, presence of maxillary canines darker than shade A3.5 on the VITA Classical shade guide (determined by spectrophotometry), willingness to refrain from smoking throughout the study period, and provision of written informed consent. Exclusion criteria comprised current orthodontic appliances, untreated carious lesions, pregnancy, unsatisfactory oral hygiene, presence of anterior restorations (16 anterior teeth, from second premolar to second premolar), previous endodontic treatment on anterior teeth, and severe developmental defects or intrinsic discolorations of dental tissues. All clinical procedures were performed according to the protocol described in detail in the original randomized clinical trial [29].

### 2.2. Spectrophotometric Image Acquisition

Spectrophotometric images of the buccal surfaces of maxillary central incisors and canines were acquired using a non-contact spectrophotometer (SpectroShade Micro, MHT Optic Research, Niederhasli, Switzerland). Images were retrieved from the device database at four time points: eligibility (control), baseline (pre-treatment), immediate post-treatment, and six-month follow-up. At each time point, three repeated measurements per tooth were obtained by an independent and blinded examiner following the manufacturer’s calibration and acquisition protocols. The corresponding CIE L*a*b* values were used in the original trial to calculate tooth colour difference (ΔE_00_), whiteness index (WID), and whiteness difference (ΔWI_D_), which were available for secondary analysis in the present study [29]. Although the bleaching protocols were originally allocated within a previously published randomized clinical trial, the present investigation should be understood as a retrospective secondary analysis of stored spectrophotometric images undertaken to assess translucency-related parameters.

### 2.3. Image-Based Tooth Translucency Assessment

**Figure 1:**
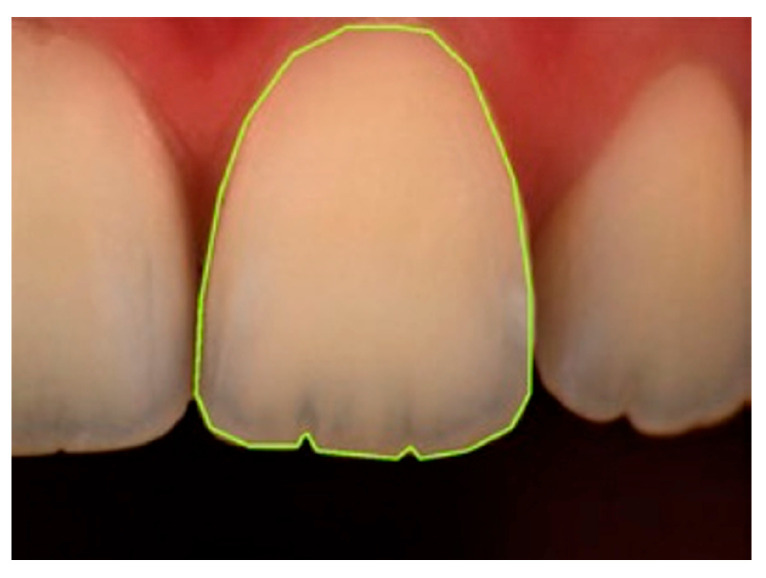
Selection of the vestibular surface in SpectroShade Analysis 2.40 software.

**Figure 2:**
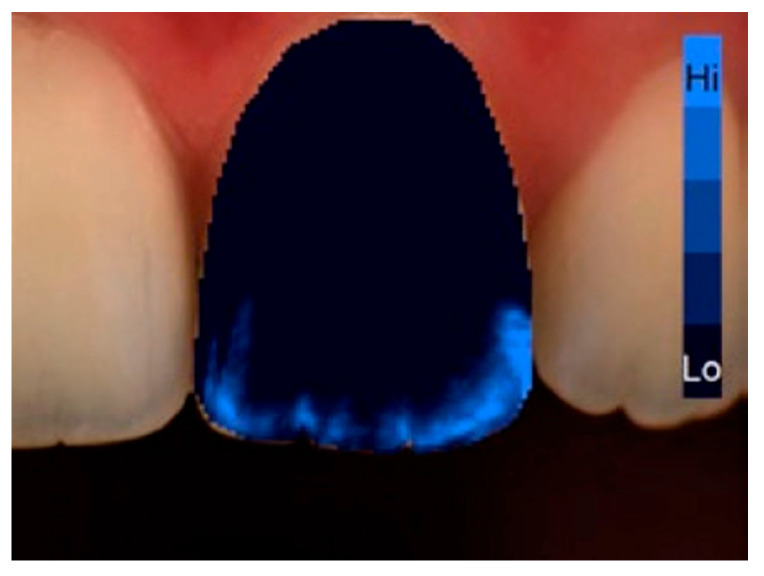
Translucency map generated in SpectroShade Analysis software.

**Figure 3:**
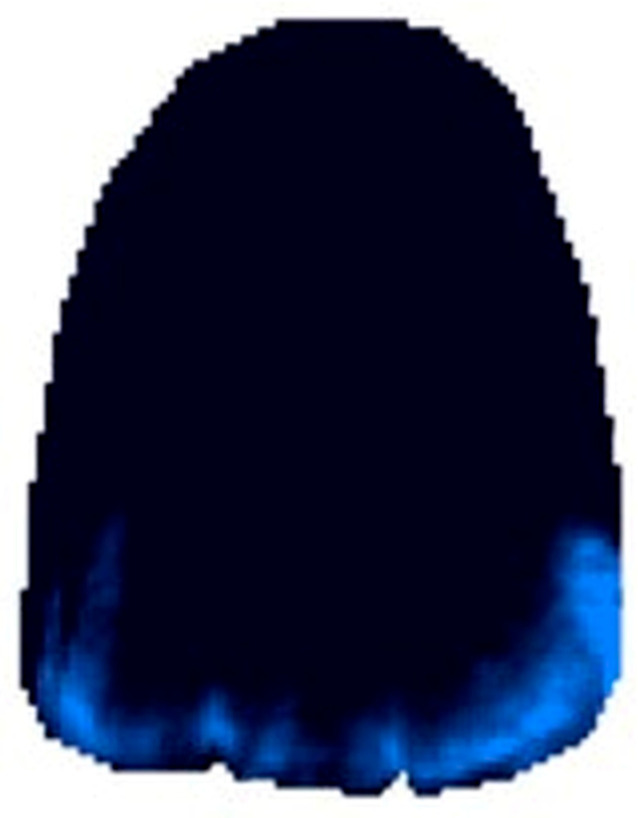
Translucency map with background removed, obtained in Microsoft^®^ PowerPoint.

**Figure 4:**
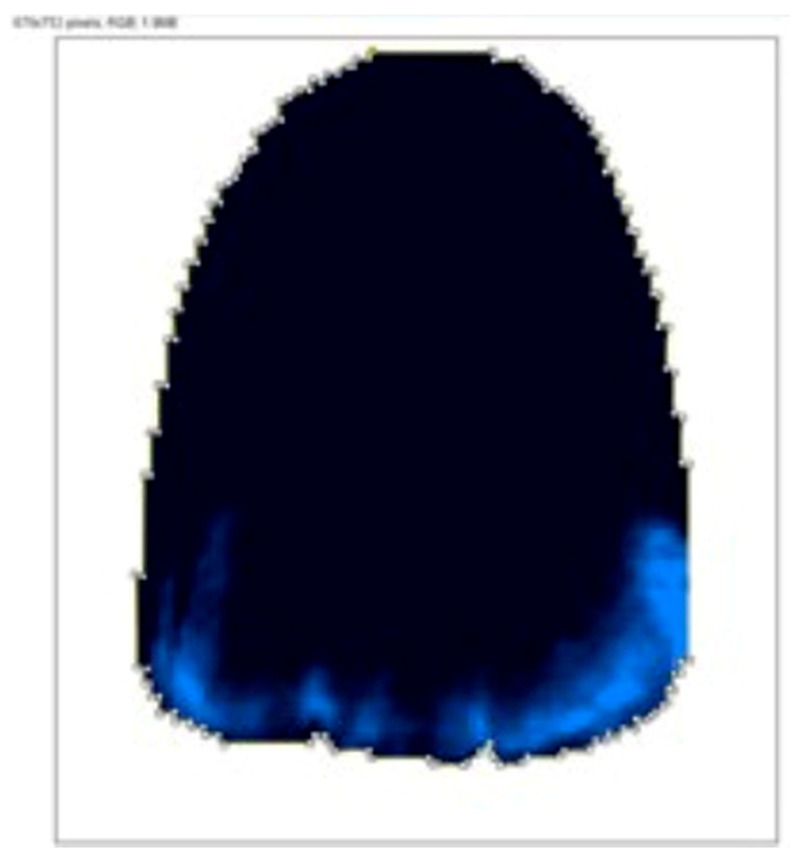
Processing of the translucency map in ImageJ, with selection of the region of interest for analysis.

**Figure 5:**
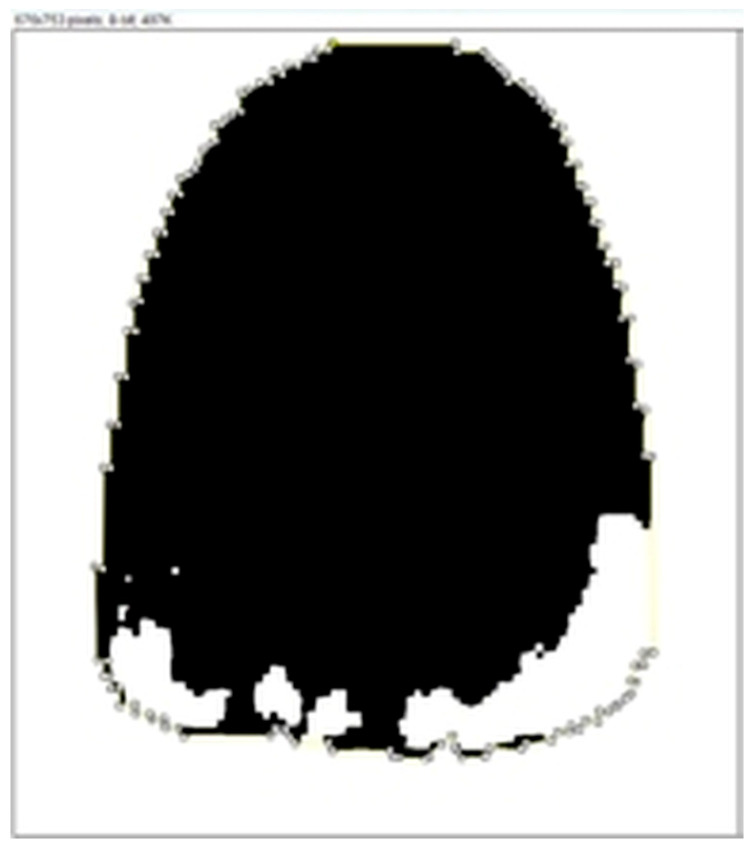
Quantification of the translucent area in ImageJ (“Analyze Particles”), following conversion of the translucency map to 8-bit image and application of “Auto Threshold—RenyiEntropy” (matching the translucency map).

**Figure 6:**
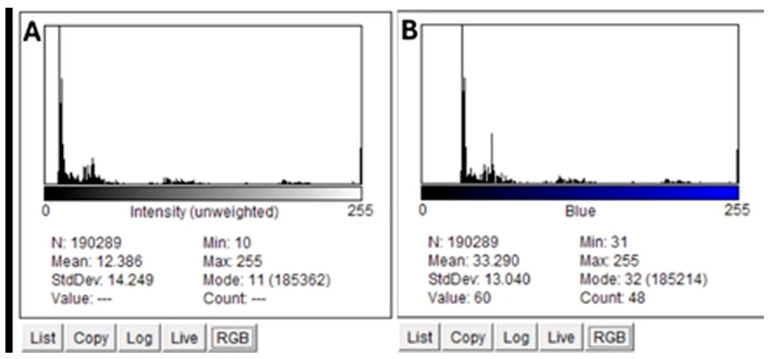
Pixel quantification in ImageJ 1.54: (**A**) histogram-derived grey intensity (0 = black/high opacity to 255 = white/high translucency); (**B**) RGB-blue channel (0 = dark blue/high opacity to 255 = light blue/high translucency).

Tooth translucency was assessed using a modified image-based methodology adapted from Todorov et al. [18]. The retrieved images of the buccal surfaces of maxillary central incisors and canines were analysed with Spectroshade Analysis software (MHT Optic Research, Niederhasli, Switzerland—Figure 1) and converted into colour-coded translucency maps (Figure 2), ranging from light blue (higher translucency) to dark blue (higher opacity). For each image, the external background surrounding the tooth was manually removed in Microsoft PowerPoint (using the remove background function) to isolate only the tooth translucency map and exclude non-relevant surrounding areas from subsequent analysis. This step was performed only to define the tooth region of interest and did not involve any intentional modification of the translucency map itself (Figure 3). The isolated translucency maps were then exported in Portable Network Graphics (PNG) and transferred to ImageJ 1.54 software (National Institutes of Health, Bethesda, MD, USA) for image processing and quantitative analysis using the “polygon selections” function within the software (Figure 4). For the analysis of the percentage of translucent area, each PNG image was opened in ImageJ and converted to an 8-bit grayscale image. Automated thresholding was then applied using the RenyiEntropy algorithm, selected after preliminary comparison with other automated thresholding methods, as it provided the most consistent segmentation of the translucency gradients observed in the spectrophotometric maps and the closest visual correspondence with the original shade distribution—available in the Image-Adjust-Auto Threshold menu. No manual threshold value was selected, as segmentation was performed exclusively through this automated thresholding procedure. After thresholding, the binary image was used to identify the pixels classified as translucent within the selected tooth region, and the percentage of the translucent area was calculated as the proportion of translucent pixels relative to the total region of interest (Figure 5). For intensity-based assessment, histogram-derived mean grey intensity and RGB-blue channel values were extracted from the isolated translucency maps without binary thresholding. Mean grey intensity was obtained from the grayscale histogram, whereas RGB-blue values were obtained from the blue-channel histogram of the original map. Both measurements ranged from 0 to 255, with lower values indicating greater opacity and higher values indicating greater translucency. These parameters were used as complementary quantitative indicators of translucency distribution and intensity across the tooth surface (Figure 6).

### 2.4. Statistical Analysis

Statistical analysis was performed using IBM SPSS Statistics version 25 (IBM Corp., Chicago, IL, USA). Data are presented as means with corresponding 95% confidence intervals (CI) for all translucency-related parameters, colour differences (ΔE_00_), and whiteness differences (ΔWI_D_). Maxillary central incisors and canines were analysed separately because these tooth types present distinct optical characteristics and potentially different responses to bleaching, making a tooth-type-specific assessment more appropriate for this pilot applicability study. To address potential within-participant clustering, a sensitivity analysis was performed using a linear mixed-effects model, with participant as a random effect and compound symmetry as the covariance structure, while different stages and groups were included as fixed effects (along with their interaction). Normality assumptions were considered acceptable based on sample size per group and the central limit theorem [30]. Intragroup differences across time points (eligibility, baseline, post-treatment, and six-month follow-up) were analysed using repeated-measures analysis of variance (RM-ANOVA) with Bonferroni adjustment for pairwise comparisons. Furthermore, sphericity was assessed using Mauchly’s test and the Greenhouse–Geisser correction was applied whenever the sphericity assumption was violated. Intergroup comparisons were performed using one-way ANOVA with Tukey post hoc tests. Associations between translucency-related parameters (percentage of translucent area, grey intensity, and RGB-blue channel values) and colour/whiteness changes (ΔE_00_ and ΔWI_D_) were assessed using Pearson’s correlation coefficient (r). Correlation strength was interpreted as negligible (0.00–0.09), weak (0.10–0.39), moderate (0.40–0.69), strong (0.70–0.89), or very strong (0.90–1.00), with moderate or higher correlations considered clinically relevant [31]. The level of statistical significance was set at α = 0.05 for all analyses.

Intraobserver reproducibility of the image-analysis workflow was assessed by the same examiner within a week interval, using the same standardized protocol, and by calculating intra-class correlation coefficient (ICC) based on a two-way mixed-effects absolute agreement model. The reproducibility of the image-analysis workflow was considered good with an ICC result of 0.75.

Given the retrospective cohort design of this study, the sample size was predefined by the dataset retrieved from the original published randomized clinical trial. A sensitivity power analysis was conducted post hoc using G*Power 3.1 software (Heinrich-Heine-Universität, Düsseldorf, Germany) to determine the minimum detectable effect sizes given the available sample size (considering a statistical power of 80% and a significance level of 5%). For the intergroup comparisons of translucency-related parameters using a one-way ANOVA, the sample size of 80 participants at the immediate post-treatment evaluation was sufficient to detect a medium to large effect size (Cohen’s f = 0.35). At the six-month follow-up, accounting for an attrition rate of 32.2%, the study maintained adequate power to detect an effect size of f = 0.41 (n = 61).

## 3. Results

Ninety participants were initially enrolled in the randomized clinical trial and descriptive data for CIE L*a*b*, WI_D_, ΔE_00_ and Δ WI_D_ were reported in the original study [29]. Due to the study period overlapping with COVID-19 quarantine restrictions, participant attrition occurred over time. Eighty participants completed the post-treatment evaluation (group A: 27; group B: 26; group C: 27), comprising 56 females and 24 males, aged between 18 and 40 years (mean age: 23.0 [22.8:23.4]). This corresponded to an attrition bias of 11.1%, whereas this value increased to 32.2% at the six-month follow-up (group A: 20; group B: 20; group C: 21). Loss to follow-up was related to COVID-19-related restrictions affecting attendance at scheduled recall visits in the original clinical trial, rather than to the intervention itself or to the present image-analysis procedures. Nevertheless, comparisons were performed between participants who completed and those who did not complete the study with respect to age, gender, randomization group, and translucency-related parameters. Continuous variables were analysed using the independent-samples t test, whereas categorical variables were analysed using Pearson’s chi-square test or Fisher’s exact test, as appropriate. No statistically significant differences (*p* > 0.05) were observed for gender, randomization group, or translucency-related parameters. However, age differed significantly (*p* < 0.05) between completers and non-completers, with non-completers being older on average—24.79 versus 22.71 years old; mean difference of 2.07 [0.40:3.75].

Translucency variation was first analysed across different time points within each group by RM-ANOVA, which revealed no significant differences (*p* > 0.05) between the control and baseline stages for any variable after Greenhouse–Geisser correction. However, post-treatment results showed a significant increase (*p* < 0.05) in all translucency-related parameters across all groups, indicating a higher tooth translucency after treatment regardless of the bleaching technique. Additionally, a significant increase (*p* < 0.05) in the percentage of the translucent area was observed at the follow-up stage (except for the incisal sample in group C). This increase was not concomitant with any significant (*p* > 0.05) changes in the histogram-derived grey intensity or the RGB-blue channel. The mean values and 95% CI of the translucency-related parameters are depicted in Table 1.

Intergroup evaluation of the translucency-related parameters was performed by using ANOVA and Tukey post-hoc, which detected significantly lower (Incisors: F = 10.0, *p* < 0.001, ηp2 = 0.12; Canines: F = 9.60, *p* < 0.001, ηp2 = 0.11) values in group A for all variables, indicating a smaller change in tooth translucency with the in-office technique, as highlighted in Table 1. These differences remained significant at the six-month follow-up (F = 7.21, *p* < 0.001, ηp2 = 0.10), except for the percentage of translucent area in incisors, which showed similar results across the different groups (F = 0.76, *p* = 0.47, ηp2 = 0.01).

To address potential within-participant clustering arising from the inclusion of two teeth per participant, a sensitivity analysis was performed using linear mixed-effects models. These models confirmed significant effects of different stages, group, and their interaction for the percentage of translucent area (F = 172.413, *p* < 0.001; F = 5.071, *p* = 0.007; F = 3.993, *p* < 0.001, respectively), histogram-derived grey intensity (F = 105.004, *p* < 0.001; F = 12.997, *p* < 0.001; F = 12.305, *p* < 0.001, respectively), and RGB-blue channel values (F = 62.706, *p* < 0.001; F = 23.135, *p* < 0.001; F = 7.004, *p* < 0.001, respectively).

The association between translucency-related parameters and differences in tooth colour/whiteness was evaluated using Pearson’s correlation coefficient, which indicated a weak positive relationship between the variables. Although some translucency-related parameters showed moderate positive correlations with post-treatment ΔWI_D_ values in groups A and B (highlighted in Table 2), most analyses did not detect significant correlations between translucency and colour/whiteness changes. For this evaluation, the ΔE_00_ and ΔWI_D_ values from the original article were considered [29]—presented in Table 3.

## 4. Discussion

This retrospective study applied and preliminarily tested a reproducible, image-based, and spatially resolved methodology for assessing tooth translucency changes following three different bleaching techniques. Using spectrophotometric translucency maps combined with quantitative image-derived analysis, the present findings demonstrated a significant post-bleaching increase in translucency-related parameters across all tested techniques. These results support rejection of the primary null hypotheses regarding immediate post-treatment translucency changes, while also revealing technique-dependent differences and limited associations between translucency and colour or whiteness outcomes. From a biomimetic perspective, this is clinically relevant because the natural appearance of teeth depends not only on colour coordinates, but also on the spatial distribution of optical properties such as translucency, which should ideally be reproduced or preserved in aesthetic procedures and restorative strategies. Nevertheless, the present findings should be interpreted within the context of a retrospective secondary analysis derived from a randomised clinical trial dataset, rather than as outcomes prespecified in the original trial.

The main finding of this study was the consistent post-treatment increase in the percentage of translucent area, histogram-derived grey intensity, and RGB-blue channel values, regardless of the bleaching protocol applied. This finding appears to contrast with most previous literature using traditional translucency parameter methods, which frequently reports minimal or inconsistent translucency changes after bleaching [24,25,26]. The discrepancy could be explained by inherent methodological limitations, which may stem from the traditional translucency assessment techniques relying on point-based measurements and failing to capture spatial heterogeneity across the tooth surface or the present map-based approach may be more sensitive to spatially distributed optical changes. Additionally, hydrogen peroxide- and carbamide peroxide-based agents are known to induce selective micro-morphological changes in enamel, including increased surface porosity, alterations in mineralisation patterns, and potential changes in organic matrix composition, which can collectively alter light-scattering characteristics in ways that an increase in tooth translucency may not be perceived due to the bulk optical density [32,33,34]. This phenomenon may underestimate translucency changes by the current translucency parameter technique, yet the map-based approach might not be affected. Moreover, the current translucency parameter formula is based on colorimetry principles and equations that may themselves impose limitations, since the translucency ratio is derived from spatial colour coordinates [12]. Nevertheless, because the SpectroShade translucency maps are generated through a proprietary reflectance-based algorithm, the exact relationship between changes in map colour distribution and visually perceived tooth translucency cannot be fully established. For this reason, increases in blue-channel intensity or threshold-based translucent area should not be interpreted as a direct surrogate of clinical translucency without further validation against conventional translucency parameters and perceptual assessments.

The present study quantified translucency not only as a percentage of translucent area but also using histogram-derived grey intensity and RGB-blue channel metrics, which were not implemented by Todorov et al. [18]. The significant post-treatment increase in both histogram parameters highlights that translucency changes involve not merely a binary classification but also a shift in translucency intensity across the mapped surface, suggesting that bleaching enhances the quantity of light transmission in a distributed manner. However, it is remarkable to observe at the six-month follow-up a continuous increase of the percentage of translucent area (in most conditions—groups A and B across both tooth types; group C incisors unchanged), whereas grey intensity and RGB-blue metrics showed no significant further change from immediate post-treatment values. This dissociation suggests that delayed translucency changes may involve redistribution of translucent regions across the tooth surface rather than further intensification of translucency within already affected areas. Post-bleaching rehydration, remineralisation, or mineral precipitation processes may contribute to this phenomenon, gradually modifying the spatial distribution of optical properties without significantly altering overall translucency intensity [32,33,34]. However, although these mechanisms may help contextualize the observed findings, they were not directly assessed in the present study and should be regarded as literature-based interpretations rather than experimentally confirmed explanations.

Intergroup comparisons revealed that the in-office bleaching protocol was associated with significantly smaller changes in translucency-related parameters compared with at-home techniques immediately after treatment and at six-month follow-up. Several clinical and procedural factors may explain this finding. In-office paint-on varnish systems are characterised by rapid peroxide release kinetics which may result in rapid substrate saturation with limited diffusion into deeper enamel layers, whereas slower diffusion profiles of at-home gels could promote more extensive enamel penetration and consequent distributed translucency changes. Furthermore, the smaller translucency changes may be partially explained by procedure-related dehydration. In-office bleaching typically involves prolonged lip and cheek retraction, continuous suction, and air exposure, which can transiently dehydrate enamel and modify its optical behaviour. Enamel dehydration is known to alter light scattering by changing the refractive index mismatch within enamel (water being replaced by air in interprismatic spaces), which may temporarily increase lightness and reduce apparent translucency, thereby masking the true post-bleaching translucency change [35,36,37,38]. Although a 30 to 45 min waiting period after post-bleaching measurements was considered by Pereira et al., evidence indicates that dehydration-induced optical changes can persist beyond short waiting intervals and that complete rehydration may require substantially longer periods [37]. Therefore, the comparatively lower translucency-related parameters changes detected in the in-office group should be interpreted cautiously, as they may partially reflect transient enamel dehydration rather than reflecting intrinsic differences in bleaching-induced optical modifications.

The weak associations observed between translucency-related parameters and colour or whiteness differences (ΔE_00_ and ΔWI_D_) challenge the assumption that increased whitening necessarily correlates with increased translucency. First, the lack of systematic covariation may reflect the multidimensional nature of optical properties associated with tooth colour perception. Bleaching mechanisms are related to the removal of chromogenic organic molecules that may increase L* and reduce a* and b*, thereby shifting the appearance toward a more neutral white [32,39]. Therefore, a tooth may become significantly whiter and lighter without major changes in translucency if the light-scattering behaviour and relative thickness of the dental tissues remain largely unchanged. This distinction is particularly relevant in biomimetic dentistry, in which the objective is not merely to obtain whiter teeth, but to preserve or reproduce the natural optical balance between enamel and dentin, including translucency-related light behaviour. Furthermore, the absence of a significant Pearson correlation does not necessarily indicate that the variables are unrelated, but rather that no evidence of a linear association was detected, as non-linear or more complex relationships may still exist [31].

Several limitations in this study must be acknowledged. First, translucency maps are derived from proprietary spectrophotometric algorithms based on reflectance modelling, which may not directly correspond to visually perceived translucency [11,14]. Second, thresholding and background removal steps introduce processing variability, even though standardised methods were applied, since the precise threshold value selected influences the resulting percentage of translucent area, and suboptimal background removal could introduce artefactual boundaries. This was mitigated by evaluating translucency-related parameters during control and baseline stages, to ensure that significant differences were related to the effect of the bleaching procedure. Third, the original dataset did not include simultaneous conventional translucency parameter measurements, thus a direct validation against established indices is not possible within this study, as it required tooth colour measurements with white and black backgrounds. Moreover, the clinical meaning of the detected differences in translucency-related image metrics remains uncertain, since no perceptibility or acceptability thresholds are currently available for this novel approach; therefore, these changes should not be interpreted as necessarily perceptible or clinically meaningful alterations in tooth translucency. Nevertheless, since the main objective was to explore the applicability of a new method, conducting a pilot study based on retrospective data was more appropriate than performing a new clinical trial. Finally, some limitations related to the sample should be acknowledged. Although the analysis was performed separately by tooth type to preserve clinically relevant optical differences, teeth from the same participant may still share subject-level characteristics. To address this potential clustering effect, additional sensitivity analyses using linear mixed-effects models were performed and showed results consistent with the primary analyses. Another point to consider is that non-completers were significantly older than completers at the 6-month follow-up, whereas no differences were observed in gender or randomization group. Therefore, although attrition was largely attributed to COVID-19-related attendance restrictions, some degree of age-related attrition bias cannot be excluded.

Future research should aim to compare and validate map-based translucency metrics with conventional translucency parameters and contrast ratios, as well as with perceptual assessments of translucency. Longitudinal studies incorporating different age groups, restorative conditions, and material interactions would further enhance understanding of bleaching-induced optical changes and support the development of standardised translucency assessment protocols. In this context, translucency assessment may also contribute to a more biomimetic approach to aesthetic dentistry, as it may help clinicians better understand whether bleaching alters optical features that are essential for reproducing the appearance of natural dental tissues.

## 5. Conclusions

Within the limitations of this retrospective study, the developed image-based and spatially resolved methodology detected significant and technique-dependent increases in translucency-related parameters following all evaluated bleaching protocols, with the in-office technique associated with smaller changes than the at-home protocols. The percentage of translucent area generally remained elevated at six months, whereas histogram-based metrics showed no further significant change after the immediate post-treatment period. However, these findings reflect variations in map-derived optical metrics from proprietary SpectroShade analysis and not direct proof of clinically perceptible translucency changes. In addition, the predominantly weak and inconsistent associations with colour and whiteness outcomes warrant a cautious interpretation, as they do not indicate a robust linear relationship, although more complex optical interdependencies cannot be excluded.

From a biomimetic perspective, these findings suggest that bleaching may alter optical features that contribute to the natural appearance of dental tissues and that objective translucency assessment may provide complementary information beyond conventional colour-based outcomes. Nevertheless, the present findings should not yet be interpreted as formal validation of the developed method. Direct comparison with established translucency assessment methods remains required and should be addressed in further studies to confirm its clinical applicability.

## Figures and Tables

**Table 1:** Mean and 95% CI of the translucency-related parameters at the eligibility (control), before tooth bleaching (baseline), after tooth bleaching (post-treatment) and six-month post-treatment (follow-up), by tooth type and bleaching technique. All groups presented significant post-treatment intragroup differences by Bonferroni-adjusted pairwise comparisons (*p* < 0.05). * Significant lower intergroup mean (*p* < 0.05).

		Percentage of Translucent Area		Histogram-Derived Grey Intensity		Histogram-Derived RGB-Blue Channel	
		Incisors	Canines	Incisors	Canines	Incisors	Canines
**Group A**	Control 27 cases n = 54	10.9 [9.7:12.0]	6.3 [5.0:7.6]	18.5 [17.6:19.5]	15.0 [14.2:15.3]	42.3 [40.8:43.8]	36.9 [35.5:37.1]
	Baseline 27 cases n = 54	9.8 [8.2:11.4]	6.7 [5.7:7.7]	17.7 [16.8:18.6]	14.5 [13.9:15.1]	40.9 [39.5:42.3]	35.9 [35.0:36.8]
	Post-treatment 27 cases n = 54	14.1 * **[12.3:16.0]**	9.5 * **[8.0:10.9]**	24.5 * **[23.0:26.1]**	16.5 * **[15.4:17.5]**	51.6 * **[49.2:54.0]**	39.0 * **[37.3:40.6]**
	Follow-up 20 cases n = 40	17.1 [13.1:21.2]	13.1 * **[9.9:16.2]**	19.8 * **[17.9:21.6]**	17.0 * **[16.0:18.1]**	44.4 * **[41.6:47.1]**	39.9 * **[38.3:41.5]**
**Group B**	Control 26 cases n = 52	10.0 [8.1:11.9]	6.3 [4.5:8.0]	17.7 [16.1:19.3]	16.1 [15.1:17.1]	41.9 [39.3:44.5]	37.9 [37.3:40.4]
	Baseline 26 cases n = 52	10.4 [8.6:12.2]	6.5 [5.3:7.8]	17.1 [15.6:18.6]	14.2 [13.5:15.0]	40.1 [37.7:42.4]	36.6 [35.4:36.7]
	Post-treatment 26 cases n = 52	18.1 [15.5:20.6]	15.1 [13.0:17.2]	26.0 [22.6:29.4]	22.4 [20.0:24.8]	53.9 [48.6:59.1]	48.2 [44.5:51.8]
	Follow-up 20 cases n = 40	22.6 [17.4:27.8]	19.4 [15.0:23.8]	24.0 [20.7:27.4]	20.7 [18.6:22.8]	50.8 [45.7:55.9]	45.5 [42.3:48.7]
**Group C**	Control 27 cases n = 54	11.3 [9.3:13.4]	5.3 [4.0:6.6]	19.1 [17.4:20.7]	15.5 [14.6:16.4]	44.1 [41.4:46.8]	38.2 [36.8:39.6]
	Baseline 27 cases n = 54	12.6 [10.7:14.5]	6.9 [5.6:8.1]	19.2 [17.4:20.9]	14.8 [13.9:15.6]	42.9 [40.2:45.6]	36.0 [34.7:37.3]
	Post-treatment 27 cases n = 54	21.1 [19.5:22.8]	14.3 [12.0:16.7]	27.6 [25.2:30.1]	22.8 [19.8:25.9]	56.3 [52.6:60.1]	48.7 [44.0:53.3]
	Follow-up 21 cases n = 42	21.8 [17.2:26.4]	16.3 [12.9:19.6]	25.3 [22.5:28.1]	21.6 [19.5:23.6]	52.8 [48.4:57.1]	46.8 [43.6:50.0]

**Table 2:** Pearson correlation (r) values between tooth colour (ΔE_00_)/whiteness (ΔWI_D_) differences and the translucency-related parameters. * Moderate correlation (r > 0.40).

		Percentage of Translucent Area		Histogram-Derived Grey Intensity		Histogram-Derived RGB-Blue Channel	
		Incisors	Canines	Incisors	Canines	Incisors	Canines
**Group A**	ΔE_00_ Baseline-treatment 27 cases/n = 54	r = −0.08	r = 0.37	r = −0.07	r = 0.36	r = −0.07	r = 0.38
	ΔWI_D_ Baseline-treatment 27 cases/n = 54	r = −0.06	r = 0.43 *	r = −0.06	r = 0.41 *	r = −0.06	r = 0.41 *
	ΔE_00_ Treatment-follow up 20 cases/n = 40	r = −0.11	r = −0.35	r = 0.33	r = −0.10	r = 0.34	r = −0.08
	ΔWI_D_ Treatment-follow up 20 cases/n = 40	r = −0.14	r = −0.16	r = 0.19	r = −0.09	r = 0.20	r = −0.04
**Group B**	ΔE_00_ Baseline-treatment 26 cases/n = 52	r = 0.36	r = 0.21	r = 0.38	r = 0.25	r = 0.38	r = 0.25
	ΔWI_D_ Baseline-treatment 26 cases/n = 52	r = 0.40 *	r = 0.29	r = 0.41 *	r = 0.33	r = 0.41 *	r = 0.34
	ΔE_00_ Treatment-follow up 20 cases/n = 40	r = 0.01	r = −0.06	r = 0.12	r = −0.32	r = 0.13	r = −0.32
	ΔWI_D_ Treatment-follow up 20 cases/n = 40	r = −0.04	r = −0.18	r = 0.03	r = −0.15	r = 0.03	r = −0.14
**Group C**	ΔE_00_ Baseline-treatment 27 cases/n = 54	r = −0.09	r = 0.27	r = −0.12	r = 0.24	r = −0.15	r = 0.23
	ΔWI_D_ Baseline-treatment 27 cases/n = 54	r = −0.06	r = 0.28	r = −0.05	r = 0.23	r = −0.07	r = 0.22
	ΔE_00_ Treatment-follow up 21 cases/n = 42	r = −0.21	r = 0.19	r = −0.02	r = 0.09	r = −0.02	r = 0.10
	ΔWI_D_ Treatment-follow up 21 cases/n = 42	r = −0.21	r = 0.20	r = −0.03	r = 0.12	r = −0.03	r = 0.13

**Table 3:** Mean and 95% CI values for ΔE_00_ and ΔWI_D_ at different times. * Statistically significant difference (*p* < 0.05) by one-way ANOVA with Tukey post hoc.

		Baseline—Post-Treatment 80 Cases			Post-Treatment—Follow-Up 61 Cases		
		Group A 27 Cases n = 54	Group B 26 Cases n = 52	Group C 27 Cases n = 54	Group A 20 Cases n = 40	Group B 20 Cases n = 40	Group C 21 Cases n = 42
**Δ** **E_00_**	**Canines**	4.0 [3.7:4.3]	4.3 [4.0:4.7]	7.7 * **[7.0:8.3]**	0.9 [0.8:1.1]	1.2 [0.7:1.7]	2.1 * **[1.7:2.4]**
	**Incisors**	2.6 [2.3:2.8]	2.4 [2.1:2.7]	4.4 * **[4.0:4.8]**	1.1 [0.9:1.3]	1.0 [0.7:1.2]	1.7 * **[1.4:2.1]**
**Δ** **WI_D_**	**Canines**	11.0 [10.0:11.0]	11.8 [10.7:12.9]	21.1 * **[19.6:23.4]**	1.3 [1.0:1.6]	1.7 [1.1:2.2]	4.5 * **[3.6:5.3]**
	**Incisors**	5.8 [4.9:6.6]	5.7 [4.9:6.4]	10.7 * **[9.5:12.0]**	1.4 [1.1:1.8]	1.3 [0.9:1.7]	2.5 * **[1.9:3.1]**

## Data Availability

Data is contained within the article.
